# Introducing Critical Pain-related Genes: A System Biology Approach

**DOI:** 10.32598/bcn.9.10.310

**Published:** 2019-07-01

**Authors:** Mostafa Rezaei Tavirani, Sina Rezaei Tavirani, Mohammad-Mahdi Zadeh-Esmaeel, Nayeb Ali Ahmadi

**Affiliations:** 1. Proteomics Research Center, Shahid Beheshti University of Medical Sciences, Tehran, Iran.

**Keywords:** Drug, Database, Pain, Gene, Network

## Abstract

**Introduction:**

Pain is valuable in diagnosis and also warning of the patients. Many molecular reagents are introduced which are related to pain. In this research, the pain-related genes are screened to identify the critical ones.

**Methods:**

First, the pain-related genes were pulling out from the STRING database, and Cytoscape software was used to make the interactome unit. Then the central genes and their neighbors were analyzed. Finally, the genes were clustered, and the essential genes were introduced.

**Results:**

After analyzing 159 genes of the network, FOS, IL6, TNF, TAC1, IL8, and KNG1 were identified as the essential genes. Further analysis revealed that 88 genes are directly connected to the central genes. More resolution led to ignoring TNF and IL8 and considering SCN-alpha and PAICS as additional critical nodes.

**Conclusion:**

Six critical genes related to pain were identified. They can be potentially considered as new drug targets. Further investigation is required to introduce the central genes as a pain killer.

## Highlights

- New possible drug targets (genes) are introduced for different types of pain.

## Plain Language Summary

Different types of pain as distressing experiences usually are associated with tissue injuries or damages. Several painkillers are introduced that each is characterized by beneficial or aversive effects. In this study, new drug targets are investigated to examine new painkillers. Among many candidates, six genes are determined, which may be suitable drug targets.

## 1. Introduction

Despite many investigations on the types of pain, unfortunately, the concept of pain is still unclear. This problem has resulted from the absence of distinction between pain sensation and its causes. Pain is a sensation, and this sense has several common features with the other sensations such as itching. It also occurs in various locations of the body. Due to this complexity, types of pain are categorized based on location, etiology, intensity, duration, and pathophysiology. However, pain cannot be considered just a physical entity but a sensation as well. Types of pain are known as distressing experiences, which are associated with damage or injury to the tissues.

Pain has various dimensions, such as sensory, emotional, cognitive, and social (Brodal, 2017; Orr, Shank, & Black, 2017; Williams & Craig, 2016). Based on the evidence, genetics has an important impact on pain sensation. So far, several genes have been introduced that affect pain sensation and its intensity. Reportedly, people respond differently to painful stimuli. Genetics may provide reasons for such different reactions between patients. Investigation shows that pain development is predictable. It seems that better understanding of the molecular mechanism of pain can mark suitable drug targets and more potent pain killers (Fillingim, Wallace, Herbstman, Ribeiro-Dasilva, & Staud, 2008; Foulkes & Wood, 2008). This genetic variability in individuals calls for continuous efforts to achieve effective treatment of pain. Genome-wide investigation and bioinformatics can be used to gain a new perspective about types of pain (Clarke et al., 2015; Smith et al., 2018).

Several therapeutic guidelines for types of pain based on reported phenotypes have been established. Some efforts are made to collect dispersed documents to present a gene set responsible for variation in pain sensation (Van Hecke et al., 2015; Veluchamy, Hébert, Meng, Palmer, & Smith, 2018). Protein-Protein Interaction (PPI) network has drawn the attention of scientists to solve such genetic complexity, which hopefully explains the types of pain. In this approach, the all known genes related to specific diseases are included in an interacting unit while each plays different roles relative to the others in the integrity of the constructed network. The important genes are highlighted as central genes and can be considered as prominent genes in the onset or development of the disorder (Safari-Alighiarloo, Taghizadeh, Rezaei-Tavirani, Goliaei, & Peyvandi, 2014). In the present study, a network analysis approach for pain is planned to introduce critically involved genes in different types of pain.

## 2. Methods

STRING database (Szklarczyk et al., 2016) was used as genes resource. The genes related to pain were extracted via disease query of the database. The genes were included in a PPI network by using Cytoscape software version 3.6.0 (Tavirani et al., 2018). Network analyzer a plugin of Cytoscape software was applied to determine the central nodes of the network. The top nodes (the nodes with closeness above mean +2 SD) were identified (Safari-Alighiarloo et al., 2017). A sub-network, including the central nodes and their direct neighbors, was constructed, too. A combination containing the least number of central nodes that were necessary to maintain the integrity of this sub-network was identified. To consider the other nodes which were not included in the neighbor nodes sub-network, they interacted in the non-neighbors nodes sub-network, and the critical nodes based on degree value were identified. The central nodes of both sub-networks were considered as key genes related to pain.

## 3. Results

**Figure 1.**
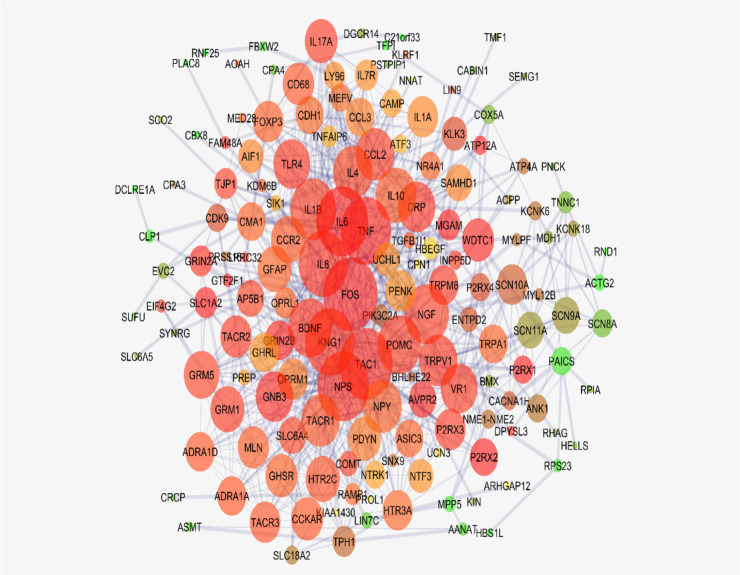
The main connected component; nodes are laid out based on degree value The main connected components of the pain network, including 159 nodes, are presented. The nodes are laid out based on degree value. Colors from green to red refer to increment degree. The bigger size refers to more connections.

A total number of 214 genes related to types of pain were extracted and included in the interactome. The network of 49 isolated genes, 2 triple units, and a main connected component (characterized by 159 nodes) was constructed (Figure 1). Closeness is defined as 1/avg L(n, m), where avg stands for average and L(n, m) is the length of the shortest path between two nodes n and m, so it is a measure of how fast information spreads from a given node to other reachable nodes in network (Malhotra, Jha, Singh, & Pandey, 2017). Therefore the nodes with the higher value of degree (or higher amount of neighbors) can be considered as the high score nodes based on closeness value. The six top nodes (FOS, IL6, TNF, TAC1, IL8, and KNG1) based on closeness value considering the cut-off value of Mean±SD were determined and presented in Table 1. In this Figure, degree, length of the shortest path between two nodes n and m, betweenness centrality, and closeness centrality related to the six central nodes are shown.

**Table 1.** The top nodes of the main connected components of the pain network based on closeness value considering cut-off values of Mean±SD

**R**	**Display Name**	**Degree**	**Stress**	**Avg (L [n, M])**	**BC**	**CC**
1	FOS	51	22700	1.91	0.13	0.52
2	IL6	47	20440	1.92	0.10	0.52
3	TNF	45	17868	1.94	0.08	0.51
4	TAC1	49	17026	1.96	0.07	0.51
5	IL8	43	14236	1.98	0.07	0.50
6	KNG1	44	14580	2.00	0.08	0.50

L (n, m). length of shortest path (distance) between two nodes n and m; BC. Betweenness centrality; and CC. Closeness centrality

**Figure 2.**
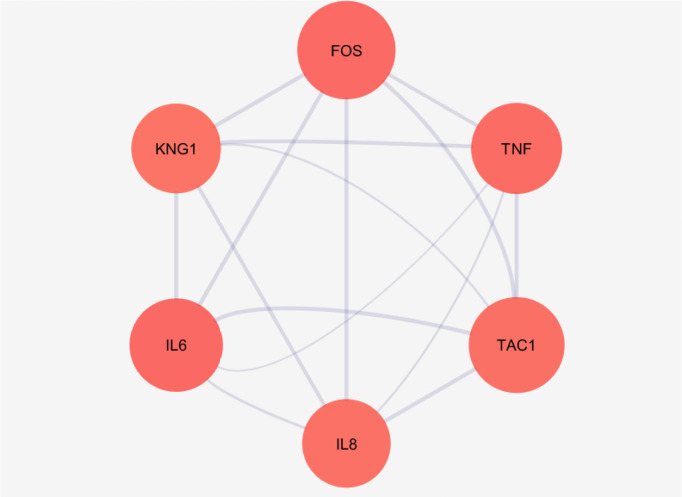
A sub-network including the 6 top nodes

**Figure 3.**
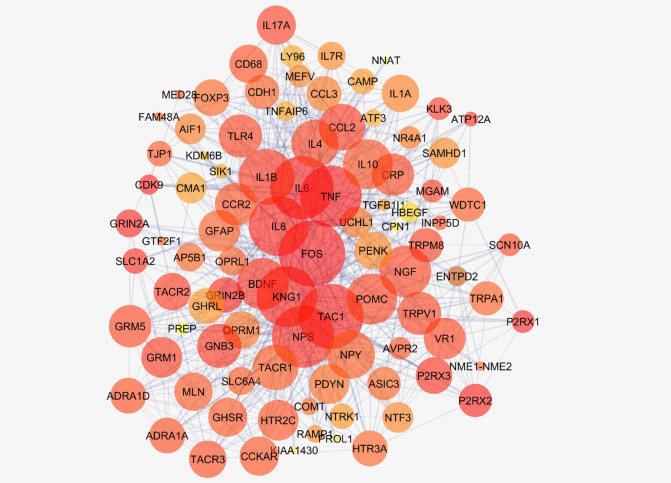
A sub-network containing 6 top nodes and their direct neighbors. A sub-network of the main connected components of the pain network containing the 6 top nodes and their direct neighbors is presented. This sub-network includes 94 nodes. Sixty-five nodes are deleted from the main connected components.

**Figure 4.**
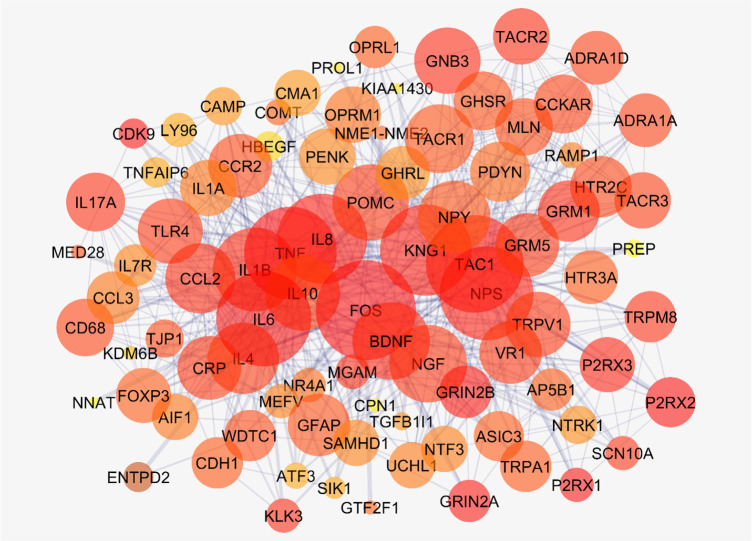
A sub-network including FOS, IL6, TAC1, and KNG1 combination and their direct neighbors A sub-network of the main connected components of the pain network, including FOS, IL6, TAC1, and KNG1 combination and their direct neighbors is presented. The sub-network included 88 nodes which its layout is based on degree value.

Figure 2 shows the connections between the six top nodes of the pain network in the represented sub-network. Considering the dense relationship between these central nodes, it seems these 6 genes and their direct neighbors play a critical role in the integrity of the pain network. A sub-network, including the 6 central nodes and their direct neighbors is constructed and is shown in Figure 3. Since the six central nodes have direct access to the 88 nodes, we examined the least number of central nodes which are necessary to maintain the integrity of the sub-network. After using different combinations of the six genes, it was found that FOS, IL6, TAC1, and KNG1 combination is the best grouping which serves as the core of the sub-network (Figure 4).

**Figure 5.**
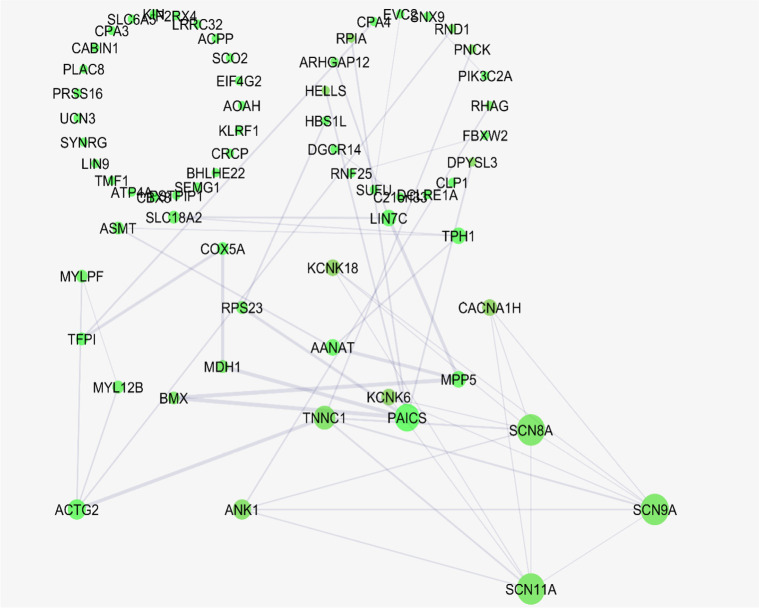
A sub-network of the nodes which are not direct neighbors of six central nodes A sub-network is represented containing the nodes which are not direct neighbors of six central nodes of the main connected components. The nodes of sub-network are laid out based on degree value. The bigger size of the node corresponds to a higher value of degree.

This sub-network includes 88 nodes (2 excluded central node+86 direct neighbors). In this procedure, the number of nodes which were not included in Figure 3 was not considered. However, these vanished genes may play roles in types of pain. So the missing genes were included in a sub-network as it is shown in Figure 5. Sub-network analysis revealed that SNC8A, SNC9A, SNC11A, (SNCA gene family) and PAICS are the hubs based on the cut-off value of Mean±SD. Mode of regulation of the 4 central nodes and SNCA gene family was investigated and shown in Table 2.

**Table 2.** Mode of regulation of the introduced central nodes

**R**	**Gene**	**Expression**	**Reference**
1	FOS	Up-regulated	(Harris, 1998)
2	IL6	Up-regulated	(Zhang & An, 2007)
3	TAC1	Up-regulated	(Shanley, Lear, Davidson, Ross, & MacKenzie, 2011)
4	KNG1	Up-regulated	(Reyes-Gibby et al., 2018)
5	SCN	Up-regulated	(Drenth & Waxman, 2007)
6	PAICS	Not determined	-

## 4. Discussion

Based on the evidence, the genes related to specific diseases need additional relevant genes to construct a scale-free PPI network. Network analysis can screen the interacted genes to find important ones which may be useful in disease management (Safari-Alighiarloo et al., 2014). As it is shown in Figure 1 and Table 1, six central nodes are introduced, which can be considered as the key elements in pain. The findings that are presented in the figures 2, 3, and 4 refer to the significant role of FOS, IL6, TAC1, and KNG1 among the six central genes in control of pain PPI network.

Further analysis (Figure 5) also revealed the noteworthy role of SNC8, SNC9, SNC11A, and PAICS in pain. It is well-known that SNC8A, SNC9A, and SNC11A are varieties of voltage-gated channel alpha subunit (SNCA) (England & De Groot, 2009). It seems that FOS, IL6, TAC1, KNG1, SNCA, and PAICS are the crucial genes related to the types of pain. As it is shown in Table 2, all crucial genes are up-regulated (a direct statement about the regulation of PAICS was not found in the literature). The possible roles of the introduced critical genes in pain are discussed briefly in the following paragraphs.

Emma V. Bird et al. investigation about the role of Chemokine Lymphotactin (XCL1) and its receptor (XCR1) in the nervous system led to introduce the role of FOS in pain. They reported that expression of c-FOS in trigeminal subnucleus caudalis exposed to XCL1 increased. Based on their analysis, XCL1-XCR1 axis is involved in the peripheral and central trigeminal pain pathway. In other words, induction of c-FOS, extracellular signal-regulated kinase, and PP38 expression and also hyper-excitability within the trigeminal subnucleus caudalis nociceptive circuitry compose the possible proposed mechanism (Bird et al., 2018).

The second central gene is interleukin 6. It is a well-known cytokine which plays different roles in host defense because of its vast range of activities in immune and hematopoietic processes. It is considered as one of the key elements in acute phase response induction. Many diseases are associated with over-expression of IL-6 (Simpson, Hammacher, Smith, Matthews, & Ward, 1997). Zhou et al. (2016) published a review of the regulatory role of IL6 in pathological pain. Based on numerous documents, they stated the essential part for IL6 in pathological types of pain. They investigated the importance of IL6 in the pathological types of pain associated with several diseases such as bone cancer, the peripheral neuropathy due to chemotherapy, and spinal cord and peripheral nerve injuries (Zhou et al., 2016).

Tachykinins encode small polypeptide chains which are known as different neuropeptides. TAC1, TAC3, and TAC4 belong to this gene family. It was established that TAC1 contributes to inflammation and immune response (Botz et al., 2013). Reportedly, TAC1 encodes neuropeptide Substance P (SP) which is involved in the neurogenic inflammation process. Up-regulation of SP in A and B neurons after noxious stimulation and its presence in C fibers indicate the role of TAC1 gene product in inflammatory pain (Shanley, Lear, Davidson, Ross, MacKenzie, 2011).

There is evidence that bradykinin is involved in sensitizing nervous (nociceptor) peripheral terminals, which lead to a reduction of pain threshold (Wang et al., 2005). The investigation is shown that there are two homologous copies; KNG1 and KNG2 as kininogen gene which High-molecular-weight Kininogen (HK) is a product of KIN1-derived mRNA. Bradykinin results from cleaved HK (Yang et al., 2017). Absence of pain due to SCN9A mutation and also a spectrum of human genetic pain disorders were reported (Drenth & Waxman, 2007; Rajasekharan, Martens, Domingues, & Cauwels, 2017). It seems that the introduced critical genes alone or in a combination are suitable candidates for drug targeting.

Network analysis showed six critical genes which are involved in pain development. Considering up-regulation of the key genes, suitable inhibitor reagents of the introduced important genes can be attentive as painkillers.
